# Prevention of Pitting in Small-Pox

**Published:** 1859-08

**Authors:** T. S. Denny

**Affiliations:** Atlanta, Georgia


### ARTICLE V.

*Prevention of Pitting in Small Pox.* By T. S. DENNY, M. D., Atlanta, Georgia.

The article in the May number of your journal, on the application of Glycerine, to prevent disfigurement from Variola, brings to my mind an instance of Varioloid, in which *pitting*, (with the exception of two or three, which might well have been taken for the slight marks frequently made by continual picking at an ordinary pimple,) was entirely avoided, by the individual opening the pustules immediately after maturity.

The patient had become convalescent enough to be left alone during the meal times of the family, and took those opportunities to procure a mirror and needle, and succeeded in opening the pustules, and removing the contents. Had the individual herself not told me of the fact, (and I have not the remotest reason for doubting it,) I certainly should never have known that she had passed through the disease. The case had been a severe one, and the eruption quite crowded on the face. I should not hesitate to recommend that course in either disease, in preference to using any application yet suggested.

The following I take to be a rational explanation of the exemption from pitting by the above means. The absorbents not being called into activity for the removal of the pus, are not liable to that excessive action which might result in the removal of a portion of the structure of the part and a consequent cicatrix.

